# One-step removal of hexavalent chromium in wide pH range using thiourea dioxide: the role of reactive species†

**DOI:** 10.1039/d3ra00520h

**Published:** 2023-04-04

**Authors:** Bin Lei, Chaoyang Wang, Ran Zhang, Zhiyong Xue, Feifei Chen

**Affiliations:** a Hubei Key Laboratory of Biomass Fibers and Eco-Dyeing and Finishing, Wuhan Textile University Wuhan 430073 Hubei China chff1977@163.com +86-27-59367336 +86-27-59367336; b College of Chemistry and Chemical Engineering, Wuhan Textile University Wuhan 430073 Hubei China

## Abstract

One-step removal of hexavalent chromium in a wide pH range is of great significance. In this paper, a single thiourea dioxide (TD) and two-components thiourea dioxide/ethanolamine (MEA) were used as a green reducing agent for the efficient removal of Cr(vi), respectively. The reduction of Cr(vi) and the precipitation of Cr(iii) were carried out simultaneously under this reaction system. The experimental results proved that TD was activated by amine exchange reaction with MEA. In other words, MEA promoted the generation of an active isomeride of TD by changing the equilibrium position of the reversible reaction. After adding MEA, the removal rate of Cr(vi) and total Cr could reach industrial water discharge standards in a wide pH range of 8–12. The change of pH, reduction potential and the decomposition rate of TD were investigated in the reaction processes. Meanwhile, reductive and oxidative reactive species were produced simultaneously during this reaction process. Further, oxidative reactive species (O_2_˙^−^ and ^1^O_2_) were beneficial for the decomplexation of Cr(iii) complexes and the formation of Cr(iii) precipitation. The experimental results also demonstrated that TD/MEA was effective in practical industrial wastewater. Hence this reaction system has a significant industrial application prospect.

## Abstract 2

The reduction/precipitation of Cr(vi) could occur simultaneously under alkaline conditions in the presence of thiourea dioxides as reducing agents.

## Graphical abstract

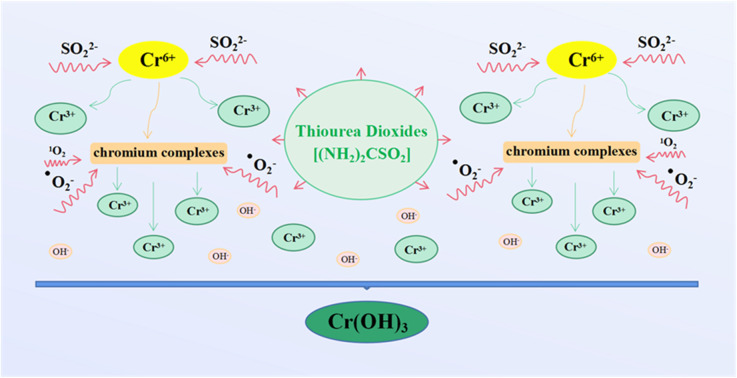

## 1. Introduction

Chromium is extensively used in industries such as electroplating, leather tanning, textile printing and dyeing, and metal processing.^1,2^ The inevitable discharge of chromium may cause environmental problems and pose a threat to human health. Many countries have established strict discharge standards for chromium-containing wastewater. Chromium exists in two main forms (Cr(vi) and Cr(iii)) in the environment. Cr(vi) is highly toxic to humans, animals and plants. By contrast, Cr(iii) is considered non-toxic due to forming insoluble precipitates under alkaline conditions. Therefore, reduction of Cr(vi) to Cr(iii) is an effective way of detoxification of Cr(vi). The common reduction methods mainly include chemical reduction, electrochemical reduction, biological reduction and photocatalytic reduction.^3–6^ Among these, chemical reduction has obvious advantages, such as simple operation, high efficiency and lost cost. Hence, the most widely used in industry is still chemical reduction. Currently, various reductants, such as sodium hydrosulfite, SO_2_, H_2_S, divalent iron, zero-valent iron, *etc.*,^7–9^ were applied in laboratory or industry. However, these reducing agents may lead to additional environmental problems due to toxicity themselves or additional precipitation. Environmentally friendly reducing agent has gained much attention recently. Researchers used natural plant extracts, such as tannic acid, sugarcane molasses, epigallocatechin gallate, *etc.*,^10–12^ as reducing agent for eliminating secondary pollution. But high prices hampered large-scale industrial applications. Meanwhile, Cr(vi) exhibits strong oxidizing property (*E*^0^(HCrO_4_^−^/Cr^3+^) = 1.35 V_NHE_) under acidic conditions, hence the reduction of Cr(vi) is usually performed in acidic condition.^13^ However, the precipitate of Cr(iii) occurs under alkaline conditions. For this reason, the conventional chemical reduction treatment method of Cr(vi) is divided into two stages. The reduction reaction of Cr(vi) is favorable under acidic condition. Hence, the pH is adjusted to less than 3 before adding reducing agents. After the reduction reaction, Cr(iii) is precipitated as insoluble hydroxides by changing the pH value of solutions to 8–10. That is to say, this process is complicated and consumes a lot of acid and base. Moreover, Cr(iii) sometimes cannot be completely precipitated due to the formation of Cr(iii) complexes,^14^ which causes high total Cr in the solutions.

Recently, the researchers have provided an economic, effective methods to removal of Cr(vi) under alkaline conditions. For instance, Pan *et al.* demonstrate that the UV/sulfite reaction system is very promising for alkaline Cr(vi) remediation.^13,15^ A alkaliphilic halotolerant strain *Pseudochrobactrum saccharolyticum* LY10 is also used for the reduction of Cr(vi) under alkaline condition.^16^ However, this reduction/precipitate process is sensitive to the change of pH values of solution. An eco-friendly reducing agent for one-step removal of Cr(vi) at a wide pH is urgently required. Meanwhile, it is also hoped that the complex of Cr(iii) can be broken during the reaction process, which is beneficial to the removal of total Cr.

As a powerful reducing agent, thiourea dioxide (TD) is widely used in textile, paper and printing industry. Especially, the reducibility of TD under alkaline condition is significantly higher than that in neutral or acid condition. Strong reducing property of TD attributes to the formation of sulfoxylic acid by breaking the C–S bond under alkaline condition.^17^ The final oxidation products of TD are urea and sulfites, COD and BOD will not rise significantly. Hence, TD is considered as an environmentally friendly reducing agent. Most important, TD exhibits stable chemical property at room temperature and natural conditions, this property makes it safe to use. It is foreseeable that TD will be more widely used in the industrial field.

In this study, single TD and two components of TD and ethanolamine (MEA) are used as reducing agent for treatment of wastewater containing Cr(vi). The reduction of Cr(vi) and the precipitation of Cr(iii) occurred simultaneously in this reaction system. The influence factors including the dosage of TD and MEA, pH value on the removal of Cr(vi) are investigated. Meanwhile, reductive and oxidative reactive species are generated during the reduction process. Reactive species are probed by electron spin resonance (EPR) and quenching experiments. Oxidative reactive species are beneficial for the decomplexation of Cr(iii) complex and the formation of Cr(iii) precipitation. After one step reaction, the content of Cr(vi) and total Cr is lower than the industrial water discharge standard in a short time. In particular, this reaction takes place in wide pH range, which brings convenience to industrial application. Further, the mechanism and reaction pathways of the removal of Cr(vi) by TD/MEA are deeply discussed.

## 2. Materials and methods

### 2.1 Reagents

All reagents were obtained from Sinopharm Chemical Reagent Co., Ltd. TD was purchased from ShenHe Auxiliaries Co., Ltd.

### 2.2 Experimental sections

Batch experiments were performed to study influence factors of the removal of Cr(vi) from the solutions. The concentration of hexavalent chromium solutions were 50 mg L^−1^. The pH was adjusted to 7–13 with HCl and NaOH. After a certain interval of time, the suspensions were extracted using a syringe and filtered through a 0.45 μm PES filter, then a UV-visible spectroscope (i9 Hanon) was employed to measure the concentration of Cr(vi) at the maximum wavelength of 540 nm using the conventional 1,5-diphenylcarbazide spectrophotometric method. TD had strong reduction ability under alkaline conditions, and could reduce phosphomolybdenum yellow to phosphomolybdenum blue. The absorbance of solution was proportional to the content of TD. To reveal the decomposition rate of TD, residual concentrations of TD in solution were determined by phosphomolybdenum blue spectrophotometry method at 710 nm.^18^ The change of pH and potential were recorded during reaction process. The reduction potentials of a solution were measured by OPR/pH apparatus (PHSJ-4F). Electron spin resonance (EPR) experiments were performed on Bruker ESR A-300 spectrometer. Liquid chromatography mass spectrometry analysis was performed on Orbitrap LC/MS (Thermo Fisher Scientific). HPLC was carried out with C18 column using water/MeOH (95/5 v/v), the flow rate was 1.0 mL min^−1^. Mass spectra were recorded in the range of *m*/*z* from 50 to 750.

## 3. Results and discussion

### 3.1 Reaction factor with TD as single reducing agent

The pH value played a key role in the removal of Cr(vi) from the solutions by reduction/precipitation processes. The dominant species of Cr(vi) was CrO_4_^2−^ at alkaline pH, and CrO_4_^2−^ had relatively low oxidation ability (*E*^0^(CrO_4_^2−^/Cr(OH)_3_) = −0.13 V_NHE_).^19^ So the reduction of CrO_4_^2−^ was the critical step for the removal of Cr(vi) under basic condition.

**Fig. 1:**
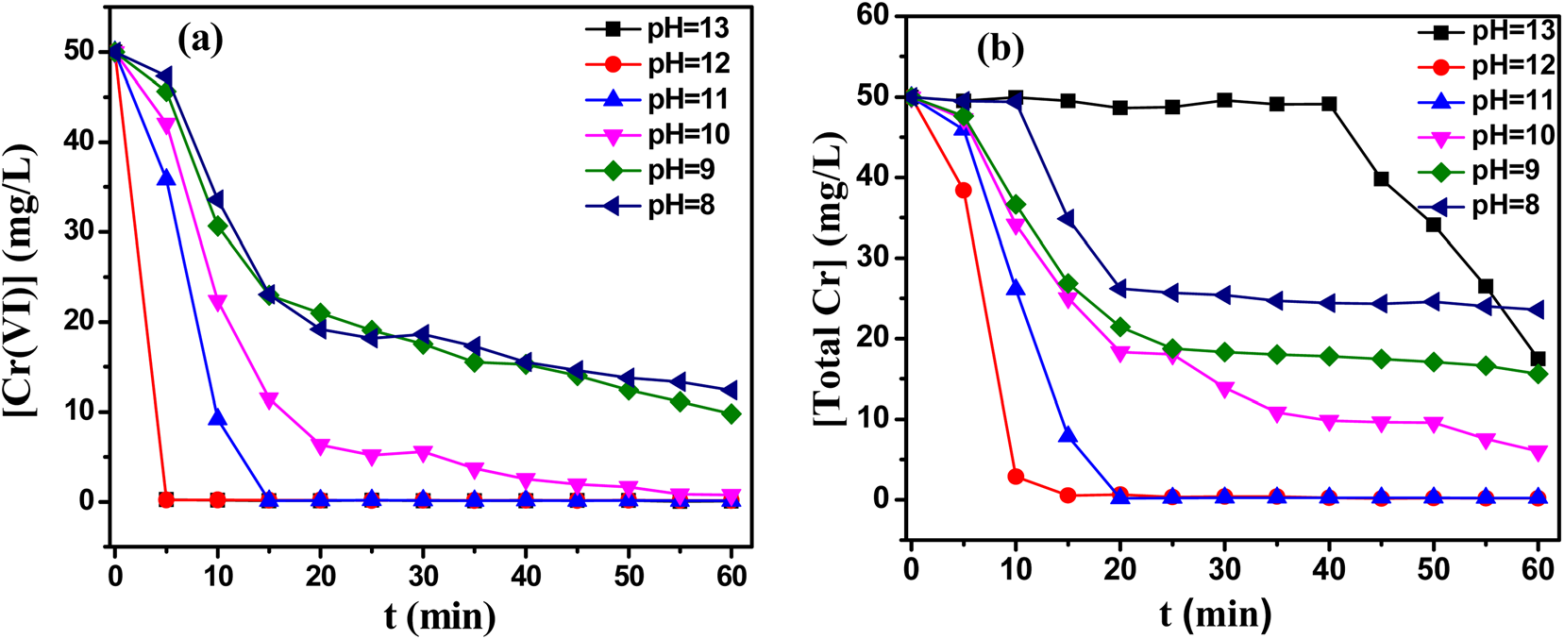
Effects of the initial pH on (a) the reduction of Cr(vi), (b) the removal of total Cr. Reaction condition: *T* = 60 °C, Cr(vi)/TD = 1 : 5.

The effects of initial pH values on the reduction of Cr(vi) and the removal of total Cr were shown in Fig. 1(a) and (b). As depicted in Fig. 1(a), the content of Cr(vi) dropped dramatically from 50 mg L^−1^ to 0.24 mg L^−1^ and 0.23 mg L^−1^ within 5 min at initial pH of 12 and 13, respectively. Moreover, the final concentration of Cr(vi) was 0.09 mg L^−1^ after 35 min at initial pH of 11. Both of them reached the industrial discharge standard of China ([Cr(vi)] < 0.1 mg L^−1^, GB 31573-2015).^20^ With the decrease of initial pH values, the reduction rate of Cr(vi) significantly decreased. The residual contents of Cr(vi) were still 6.02 mg L^−1^ and 9.77 mg L^−1^ after 60 min at initial pH of 10 and 9. This was because that the reduction potential increased with increasing alkalinity of the solution (in Fig. S1(a)†). When the initial pH was 12 and 13, the initial reduction potential increased to −822.3 and −742.1 mV quickly and maintained relatively high reduction potential. When the initial pH value decreased to 10, the highest reduction potential was −288.2 mV after 20 min, which was no enough to reduce Cr(vi). Generally speaking, the reducibility of TD depended on the decomposition rate of TD. This was because that the reducibility of TD mainly came from decomposition to produce sulfoxylate (SO_2_^2−^).^21^ As shown in Fig. S1(b),† the decomposition rate of TD increased with the increasing of alkalinity of solutions. The contents of TD decreased from 520 mg L^−1^ to 1.76 mg L^−1^ and 23.53 mg L^−1^ within 1 h under initial pH of 13 and 12, respectively. When an initial pH was less than 11, the decomposition rates of TD were obviously slower. Fig. 1(b) showed the effect of initial pH on the removal rate of total Cr. The residual total Cr in solution decreased down to 0.134 mg L^−1^ and 0.442 mg L^−1^ in 30 min at initial pH of 11 and 12, which below the industrial water discharge standard of China ([total Cr] < 0.5 mg L^−1^ or 1.0 mg L^−1^). However, beyond the pH range from 11 to 12, the residual total Cr contents were raised rapidly. For initial pH of 13, the total Cr content was still 17.48 mg L^−1^ after 60 min, even though the content of Cr(vi) was extremely low (0.072 mg L^−1^). To illustrate this experimental phenomenon, the change of pH values were investigated during reaction processes (in Fig. S1(c)†). The pH of the solution decreased due to the consumption of hydroxyl during the reduction/precipitation reaction processes. When the initial pH was less than 10, the final pH of solution reached about 4, the precipitation of Cr(OH)_3_ could not be generated under this pH value. When the initial pH value was 13, the final pH was stable around 12. The following reaction was performed at this pH value (eqn (1)), the Cr(OH)_3_ precipitation redissolved, hence the total Cr almost unchanged, the crystal of chromate was formed and the total Cr decreased until 40 minutes. However, the total Cr did not meet the industrial wastewater discharge standards after 1 h. In conclusion, the optimal pH range was 11–12 in the presence of single TD.

1 Cr(OH)_3_ + OH^−^ → CrO_2_^−^ + 2H_2_O

**Fig. 2:**
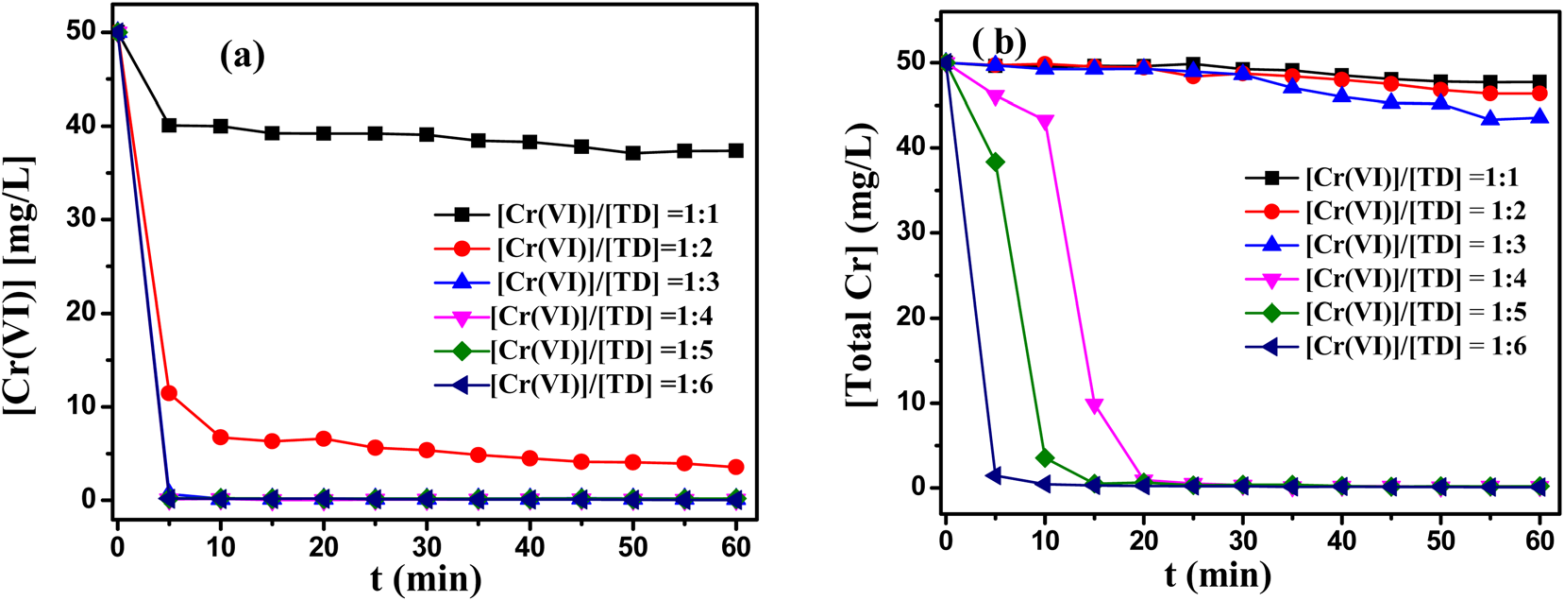
Effects of the molar ratio of Cr(vi)/TD on (a) the reduction of Cr(vi), (b) the removal of total Cr. Reaction condition: *T* = 60 °C, the initial pH value of 12, 50 mg L^−1^ Cr(vi) concentration.

The effect of TD dosage on the removal of Cr(vi) and total Cr content was investigated under 60 °C, the initial pH value of 12 and 50 mg L^−1^ Cr(vi) concentration. The dosages of TD were determined by the molar ratio of Cr(vi) to TD (from 1 : 1 to 1 : 6). The experimental results were presented in Fig. 2. When the molar ratio of Cr(vi) to TD was less than 1 : 2, the contents of residual Cr(vi) were still relatively high level. According to Fig. S2(a),† the reduction potential were very low after 10 min under the molar ratio of Cr(vi) to TD less than 1 : 2. Further, Fig. S2(b)† showed that TD was decomposed completely within 5–10 min, which indicated the molar ratio of Cr(vi) to TD was insufficient to reduce Cr(vi). So the contents of Cr(vi) and total Cr were still a high level (in Fig. 2(a) and (b)). For the molar ratio of Cr(vi) to TD = 1 : 3, the highest reduction potentials were −580 mV and the high reduction potential could be maintained about 20 min, which resulted in the high reduction rate of Cr(vi). However, the total Cr was very high, which meant that TD also played a role in the precipitation process. When the molar ratio of Cr(vi) to TD exceeded 1 : 3, Fig. 2(a) and (b) showed the content of Cr(vi) and total Cr were all less than 0.15 mg L^−1^ and 0.2 mg L^−1^ within 1 h, respectively. Fig. S2(c)† revealed that the final pH values was consisted with the optimum pH range of Cr(OH)_3_ precipitates. Especially if the molar ratio of Cr(vi) to TD was 1 : 6, the content of Cr(vi) and total Cr reached 0.038 mg L^−1^ and 0.098 mg L^−1^, which met drinking water standards of China. Considering the cost factor, the molar ratio of Cr(vi) to TD = 1 : 4 was adopted in future studies.

### 3.2 Reaction factors with two component reducing agent

Although TD had many advantages as a reducing agent, the TD–Cr reaction system still had some drawbacks. One of the main disadvantages was that the optimal pH range was relatively narrow under single TD reduction system. In order to solve problem, a second component was added to this reaction system. As shown in Table S1,† the removal ratios of Cr(vi) and total Cr were increased with adding ethanolamine (MEA), diethanolamine (DEA) and trimethylamine (TEA) as the second component. Meanwhile, considering the good biodegradability of reagent, MEA was used to improve reduction properties in the TD–Cr reaction system.

**Fig. 3:**
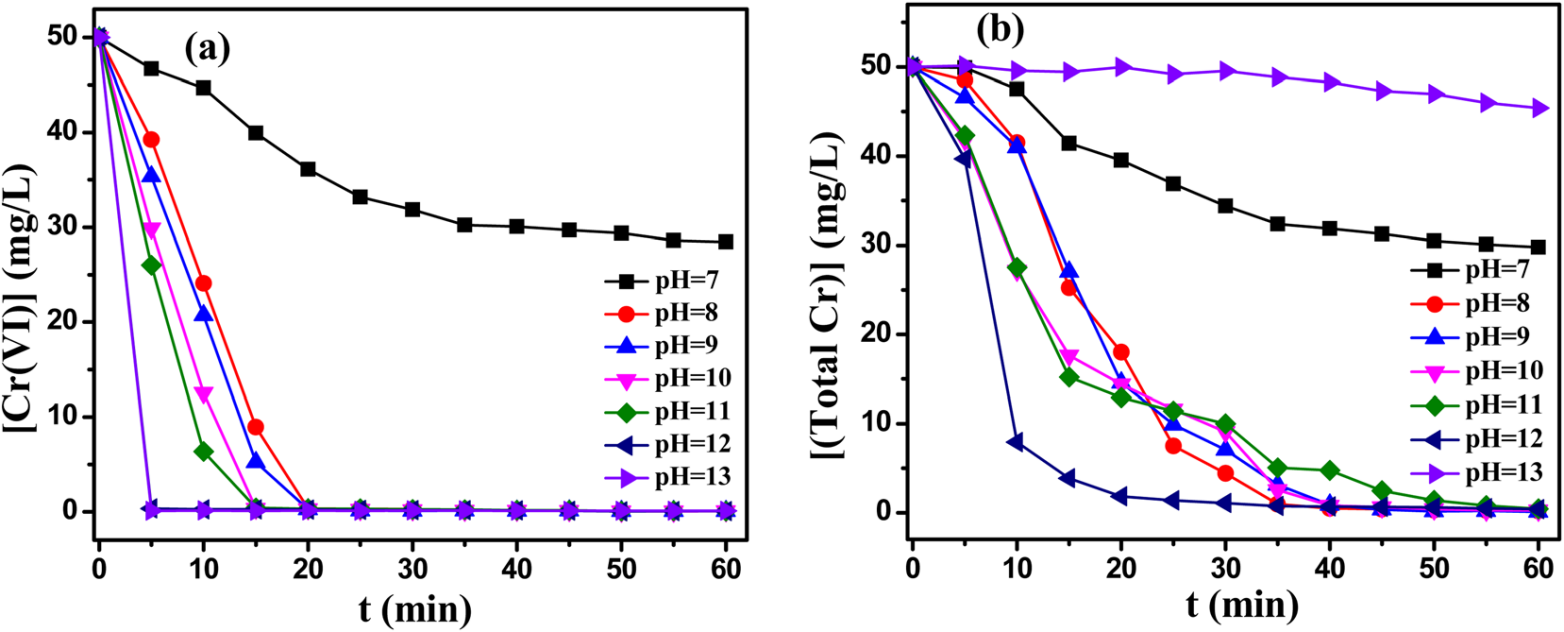
Effect of ethanolamine on (a) the reduction of Cr(vi) and (b) the removal of total Cr. Reaction condition: Cr(vi) : TD : MEA = 1 : 3 : 1, [Cr(vi)] = 50 mg L^−1^, temperature 60 °C.

TD/MEA two component reduction system was studied under various pH values. A two-component reducing agent with the molar rate of TD/MEA 3 : 1 was used in the pH range of 7–13. As depicted in Fig. 3, the removal rate of Cr(vi) and total Cr reached industrial water discharge standards in the pH range of 8–12. With the addition of MEA, the two components reduction system exhibited higher reduction potential than the single component reduction system (Fig. S3(a)†), resulting in the high reduction rate of Cr(vi). However, continue to increase pH to 12, the removal of total Cr decreased significantly (Fig. 3(b)), the reasons for this would be further investigated in future work. Compared to single component reduction system, the range of pH values extended from 11–12 to 8–12.

**Fig. 4:**
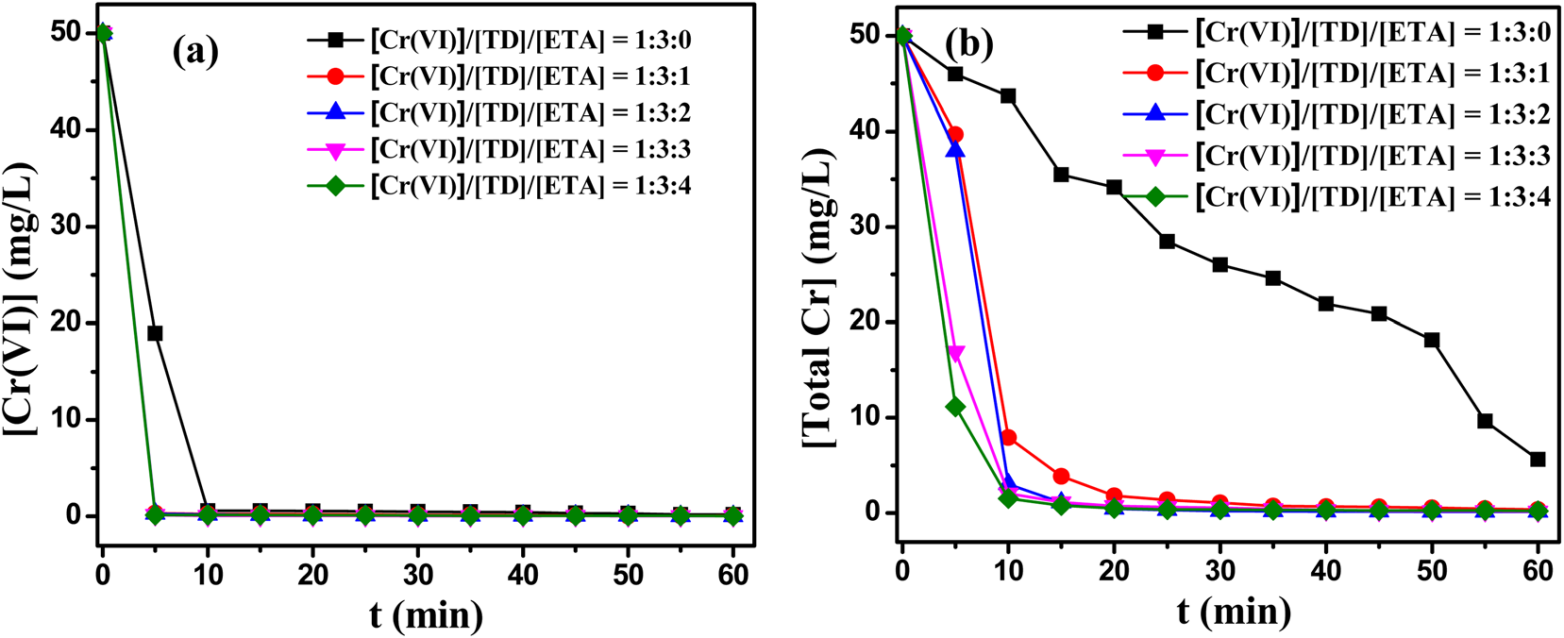
Effect of the composing proportion of TD/MEA on (a) the reduction of Cr(vi); (b) the removal of total Cr. Reaction condition: *T* = 60 °C, the initial pH value of 11, 50 mg L^−1^ Cr(vi) concentration.

The effects of the composing proportion of TD/MEA on this reduction/precipitate reaction were presented in Fig. 4. As shown in Fig. 4(a), the reduction of Cr(vi) and the removal of total Cr increased significantly with adding MEA, the all residual contents of Cr(vi) less than 0.3 mg L^−1^ in 5 min. When TD/MEA was 3 : 2, the content of Cr(vi) was just 0.01 mg L^−1^ after 1 h. Fig. 4(b) showed that the removals of total Cr were also obviously promoted after adding MEA. For single TD system, the total Cr was still higher than 5.6 mg L^−1^ even after 1 h. However, the total Cr was all lower than 1 mg L^−1^ after 30 min under reducing agent with the molar rate of TD/MEA 3 : 1. When the molar rate of TD/MEA was 3 : 2, the total Cr was only 0.2 mg L^−1^ after 30 min. Fig. S4(a)† showed that the highest reduction potential did not change with adding varying amounts of MEA. But the higher the amount of ethanolamine, the faster the peak of reduction potential was reached. In addition, the addition of MEA alone could not remove Cr and total Cr from water. The experimental results demonstrated MEA could effectively promote the reactivity of TD.

### 3.3 The possible mechanism of MEA activation of TD

**Scheme 1:**
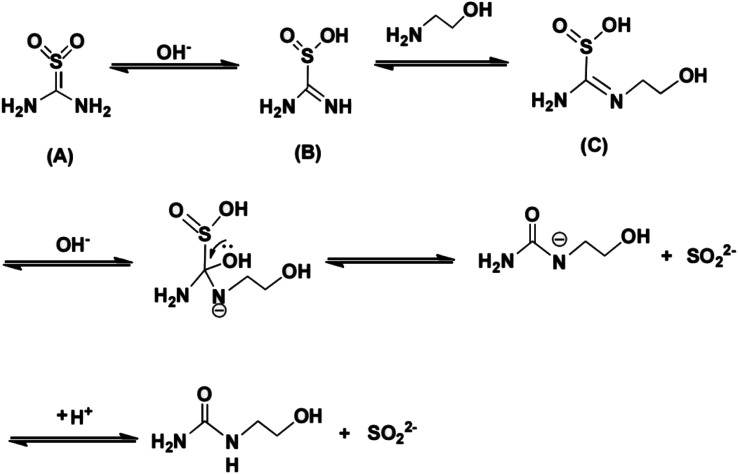
Reaction mechanism of the amine exchange reaction between TD and MEA.

TD had two isomeristic structures (A and B) (in Scheme 1) in the solution. Isomeride (A) and isomeride (B) could convert each other and reach equilibrium. Under acidic condition, TD existed in the form of isomeride (A). With the increasing of pH, the structure of TD was transformed into isomeride (B), the property of isomeride (B) was unstable and was easily converted to strongly reductive species. Therefore, TD displayed stronger reducibility under alkaline condition. Furthermore, the phenomenon suggested that the reducibility of TD should be enhanced by increasing the proportion of isomeride (B).

As discussed above, the addition of MEA could effectively improve the reduction ability of TD. The possible reaction routes are shown in Scheme 1. The imine C=N group in the isomeride (B) was considered as dynamic chemical bond, exchange reaction might easily happen.^22,23^ In this reaction system, the amine exchange reaction occurred between MEA and isomeride (B) of TD to form the intermediate (C). This exchange reaction shifted the equilibrium toward the right side, stable isomeride (A) was easier to convert into unstable isomeride (B), resulting in increasing reducibility of the reaction system. Hence, the application range of pH value was extended and the dosage of reducing agent was reduced.

**Fig. 5:**
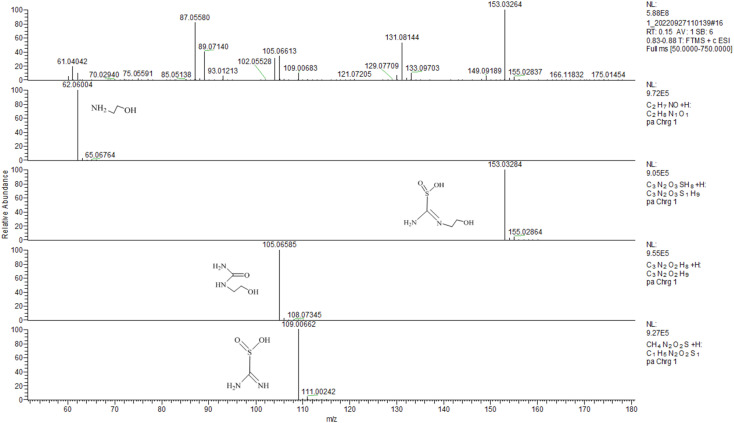
LC/MS spectrum monitored the intermediates under TD/MEA after 30 min.

To fully understand the reduction pathway in TD/MEA two-component system, the 30 min solution was analyzed to identify intermediate products by LC-MS analysis (Fig. 5). The *m*/*z* 153 corresponded to the protonated intermediate (C), this was proof that an amine exchange reaction had taken place. The *m*/*z* 105 derived from the protonated decomposition products of intermediate (C), at the same time an equal mole of SO_2_^2−^ was produced during decomposition reaction. Unreacted TD and MEA at *m*/*z* 109 and 62 were also found in the spectrum, implying that the solution still had some reducibility after 30 min. The experimental result confirmed the occurrence of amine exchange reaction.

### 3.4 Roles of major reactive species

#### 3.4.1 Reductive reactive species

Generally speaking, three reactive species, such as e_aq_^−^, H˙ and SO_3_˙^−^ might be involved in this Cr(vi) reduction process.^24^ The role of H˙ could be ignored because of the low concentration under basic condition. To verify whether e_aq_^−^ existed on this reaction system, NO_3_^−^ and monochloroacetic acid (MCAA) was selected as scavenger for e_aq_^−^ (9.7 × 10^9^ M^−1^ s^−1^ and 1.0× 10^9^ M^−1^ s^−1^, respectively).^24,25^ Both 5 mM NO_3_^−^ and 1.0 mM MCAA had little effect on the reduction of Cr(vi) (Fig. S5†), which demonstrated that e_aq_^−^ could not be produced under natural light.

The stability of TD became poor under alkaline condition, because OH^−^ acted as a nucleophilic agent to attack the carbon center, resulting the breaking of C–S bond and the formation of urea and SO_2_^2−^ (eqn (2)).^26,27^ As a strong reducing species (−1.12 to −0.74 V),^28^ sulfoxylate could reduce Cr(vi) to Cr(iii) (eqn (3)).^29^ Further, SO_3_˙^−^ was also simultaneously generated during the reduction process (eqn (4)). SO_3_˙^−^ was regarded as having mild oxidant or reductant (*E*^0^(SO_3_˙^−^/SO_3_^2−^) = 0.73 V_NHE_, *E*^0^(SO_4_^2−^/SO_3_˙^−^) = −2.47 V_NHE_). SO_3_˙^−^ could easily reduce Cr(v) and Cr(iv) to Cr(iii) (eqn (5)–(7)),^30^ which illustrated that SO_3_˙^−^ contributed to the reduction of Cr(vi) under alkaline condition and SO_4_^2−^ was the final products. We confirmed that the sulfur of TD was almost converted to SO_4_^2−^ and SO_3_^2−^.

2 (NH_2_)_2_CSO_2_ + 2OH^−^ → (NH_2_)_2_CO + SO_2_^2−^ + H_2_O

3 SO_2_^2−^ + Cr(vi) → SO_3_^2−^ + Cr(iii)

4 SO_3_^2−^ + Cr(vi) → SO_3_˙^−^ + Cr(iii)

5 Cr(vi) + SO_3_˙^−^ → Cr(v) + SO_4_^2−^

6 Cr(v) + SO_3_˙^−^ → Cr(iv) + SO_4_^2−^

7 Cr(iv) + SO_3_˙^−^ → Cr(iii) + SO_4_^2−^

**Fig. 6:**
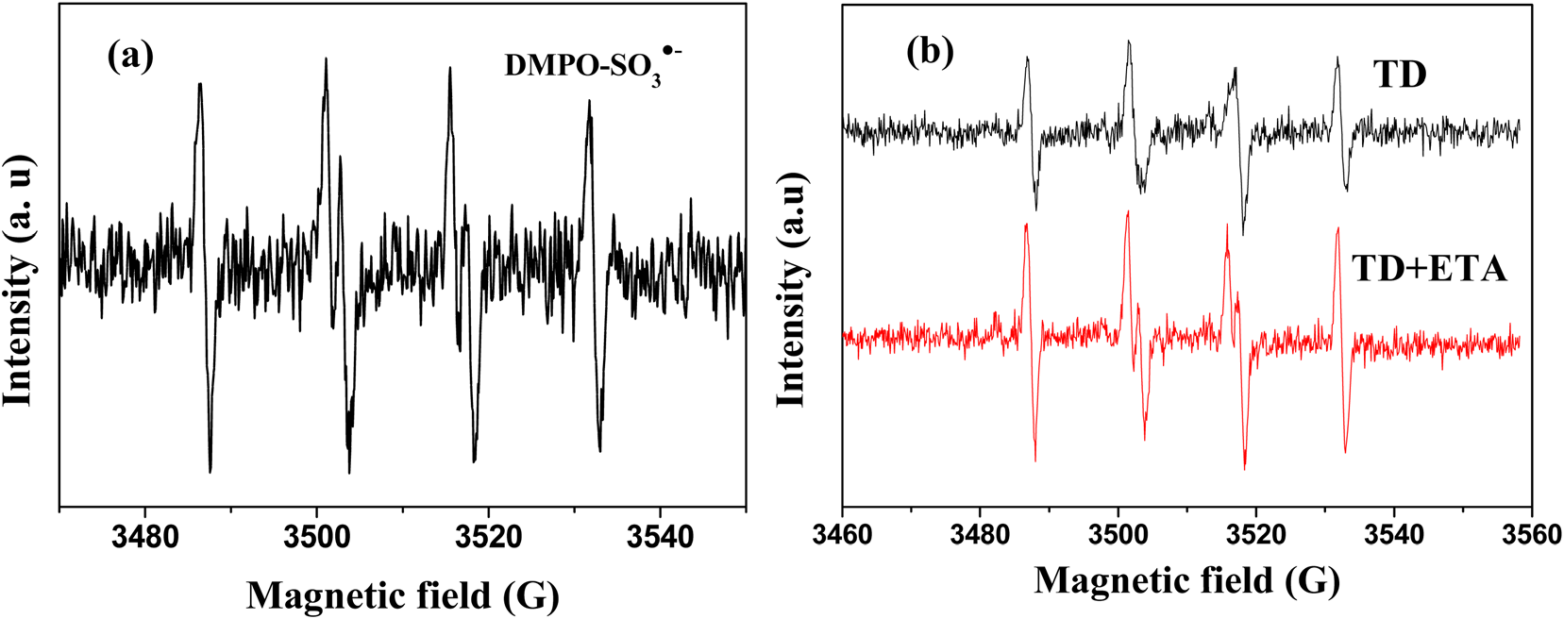
(a) Sulfite radical (100 mM DMPO, [Cr(vi)] = 1 mM, the molar ratio of Cr(vi) to TD = 1 : 4, the initial pH value of 12, reaction time 15 min); (b) EPR spectra of DMPO–SO_3_˙^−^ adduct under TD and TD/ETA (3 : 1) after 15 min.

To confirm the presence of SO_3_˙^−^, electron paramagnetic resonance (EPR) was employed. The characteristic signal of DMPO–SO_3_˙^−^ radical adduct (*a*^N^ = 14.7 G and *a*^H^ = 16.0 G) was presented in Fig. 6(a).^31^ Moreover, compared to using a single TD reducing agent, the intensity of SO_3_˙^−^ relatively higher in the presence of TD/MEA (Fig. 6(b)), which also proved that the reactivity of TD was enhanced by adding MEA.

#### 3.4.2 Oxidative reactive species

**Fig. 7:**
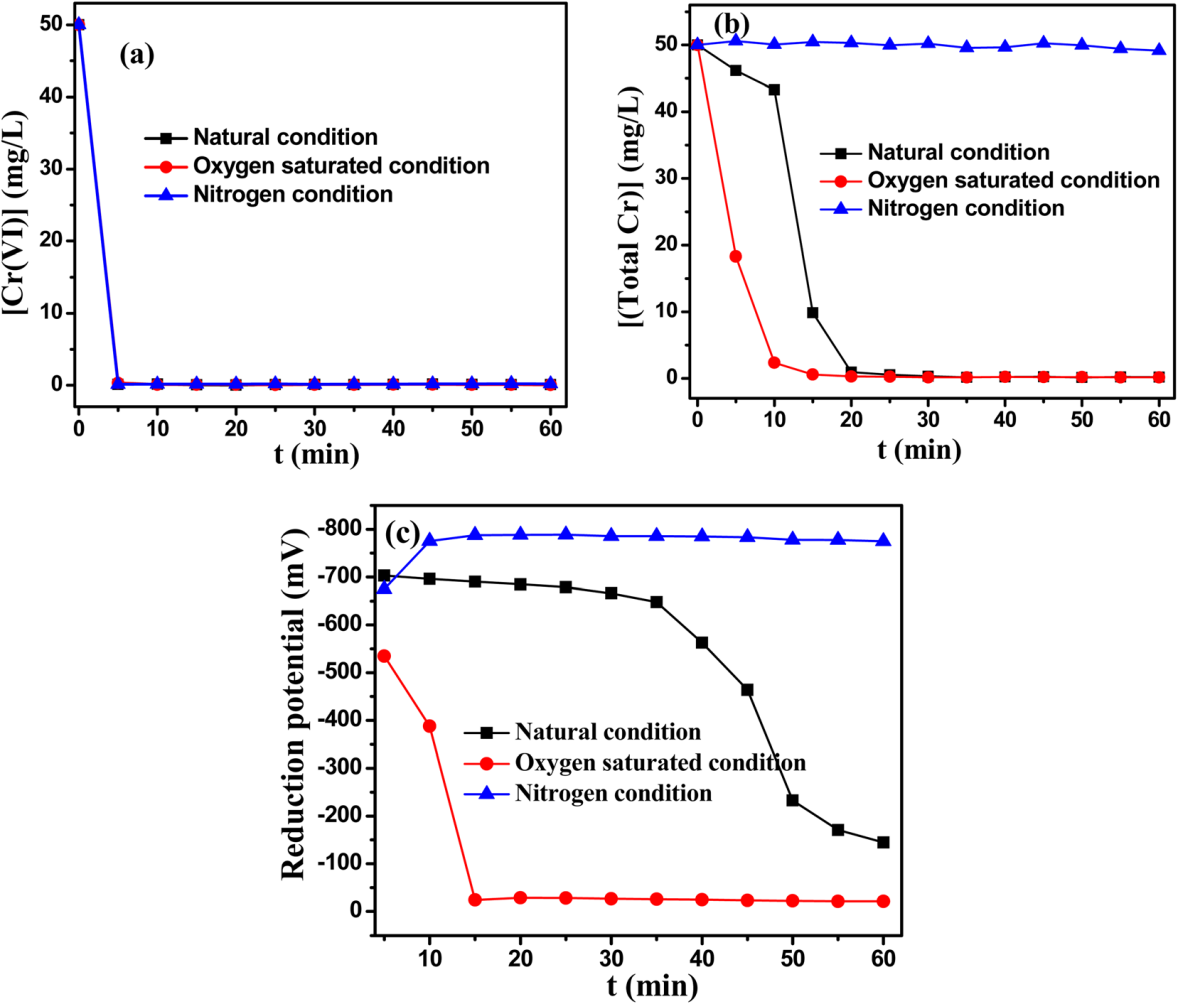
(a) The removal of Cr(vi); (b) the change of total Cr; (c) the change of reduction potential under different gas atmosphere. Reaction condition: Cr(vi) : TD : MEA = 1 : 3 : 1, the initial pH value of 12, 50 mg L^−1^ Cr(vi) concentration, temperature 60 °C.

In this study, we found that the oxygen atmosphere had a significant effect on the removal of total Cr by promoting the formation of chromium hydroxide precipitation. Therefore, it was rational to speculate that molecular oxygen might be activated to oxidative reactive species during this reaction process. To further investigate this point, the effect of oxygen concentration on the removal of Cr(vi) and total Cr was studied and the results were presented in Fig. 7. As depicted in Fig. 7(a), the reduction rate of Cr(vi) barely changed under different oxygen content. However, the removal of total Cr was very low in the atmosphere of nitrogen. The experimental results clearly showed that the precipitation of Cr(iii) were promoted in the presence of oxygen. It was because that the complex of Cr(iii) was formed in this reaction system, which hampered the precipitation of Cr(OH)_3_.^32,33^ During TD reducing Cr(vi) process, SO_2_^2−^ could be oxidized to SO_3_˙^−^ and O_2_˙^−^ in the presence of oxygen (eqn (8)).^34^ O_2_˙^−^ could be further converted to ^1^O_2_ (eqn (9)).

8 SO_2_^2−^ + O_2_ → SO_3_˙^−^ + O_2_˙^−^

9 O_2_˙^−^ → ^1^O_2_ + e

The total reduction reaction equation was presented in eqn (10). As shown in eqn (10), the theoretical moral ratio of Cr(vi)/TD was 1 : 1.5. This results also proved that the excessive consumption of TD was added in the actual reaction system, which could be ascribed to the generations of the reactive oxidants during TD reducing Cr(vi) process. Cr(iii) complex was broken in the presence of reactive oxidants, thus enhancing the precipitation of chromium ions. By contrast, aeration of nitrogen to the Cr(vi)–TD reaction system hindered further Cr(iii) precipitation.

10 3(NH_2_)_2_CSO_2_ + 2CrO_4_^2−^ + 2OH^−^ + 5H_2_O → 3(NH_2_)_2_CO + 2Cr(OH)_3_ + 3SO_4_^2−^

Fig. 7(c) displayed the change of reduction potential. As we had hypothesized, the reducibility decreased rapidly and the system had no reductive ability after 15 minutes under saturated oxygen atmosphere, this was because that reducing substances could react with oxygen molecule. However, the reduction of Cr(vi) had almost completed within 5 min, hence it had not no effect on the reduction effect of Cr(vi) under saturated oxygen atmosphere. Meanwhile, the reduction potential did not decrease even after 1 h under nitrogen atmosphere, implying that excess TD was added for the reduction process.

**Fig. 8:**
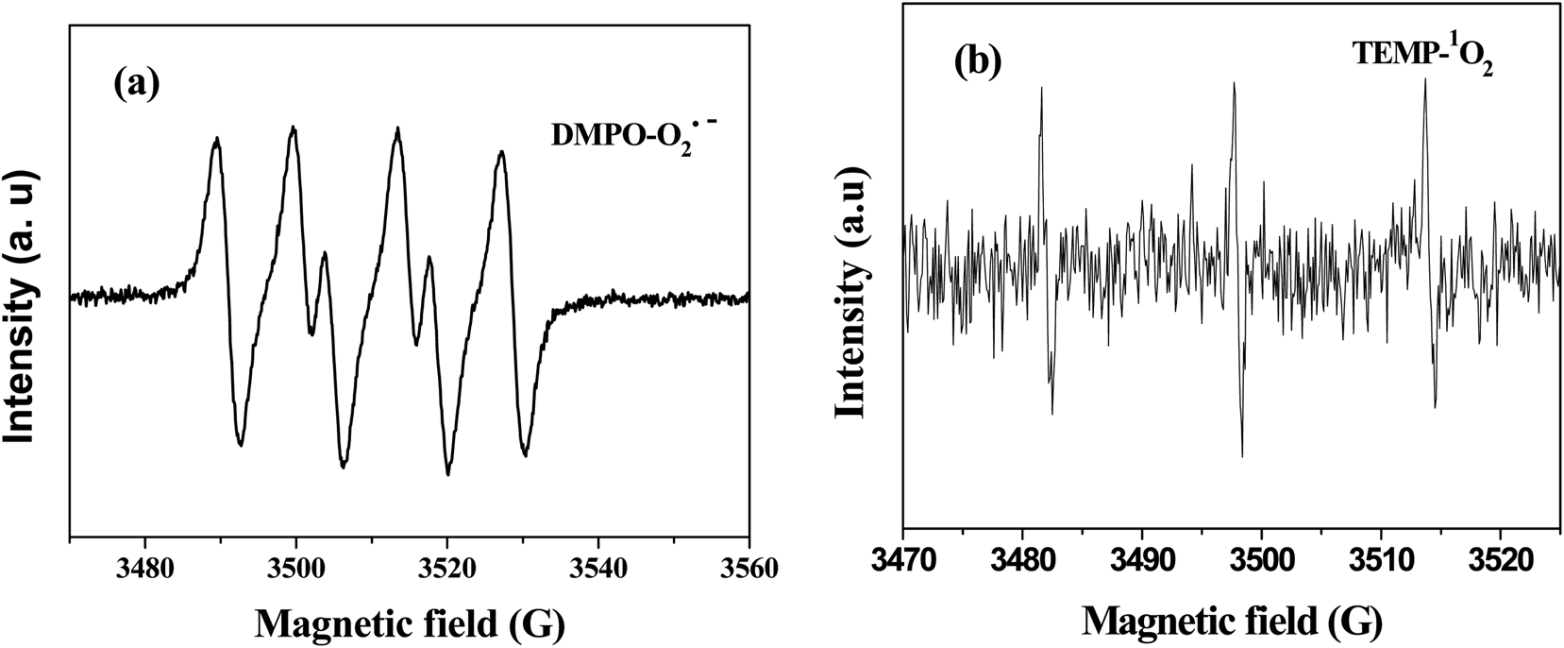
ESR spectrum of (a) superoxide radical (100 mM DMPO, [Cr(vi)] = 1 mM, Cr(vi) : TD : MEA = 1 : 3 : 1, the initial pH value of 12, reaction time 15 min); (b) singlet oxygen (100 mM TEMP, [Cr(vi)] = 1 mM, Cr(vi) : TD : MEA = 1 : 3 : 1, the initial pH value of 12, reaction time 30 min).

To verify the presence of reactive oxidants species, electron paramagnetic resonance (EPR) was employed under reaction condition. DMPO and TEMP was used as spin trapping agents for detection of reactive oxidants in water and methanol, respectively. As shown in Fig. 8(a), the signals of DMPO–O_2_˙^−^ with intensity ratio of 1 : 1 : 1 : 1 were clearly observed.^35^ The signal of TEMP–^1^O_2_ confirmed the existence of ^1^O_2_ in this reaction system (Fig. 8(b)).^36^ No the signal of OH˙^−^ was observed, which proved OH˙^−^ could not generate in this reaction system. The EPR spectra was also collected under nitrogen atmosphere, there were no signals of O_2_˙^−^ and ^1^O_2_, suggesting the reactive oxidants could not spontaneously produce without oxygen.^37^ These results confirmed the participation of oxygen for O_2_˙^−^ and ^1^O_2_ generation in the Cr(vi)–TD system.

**Fig. 9:**
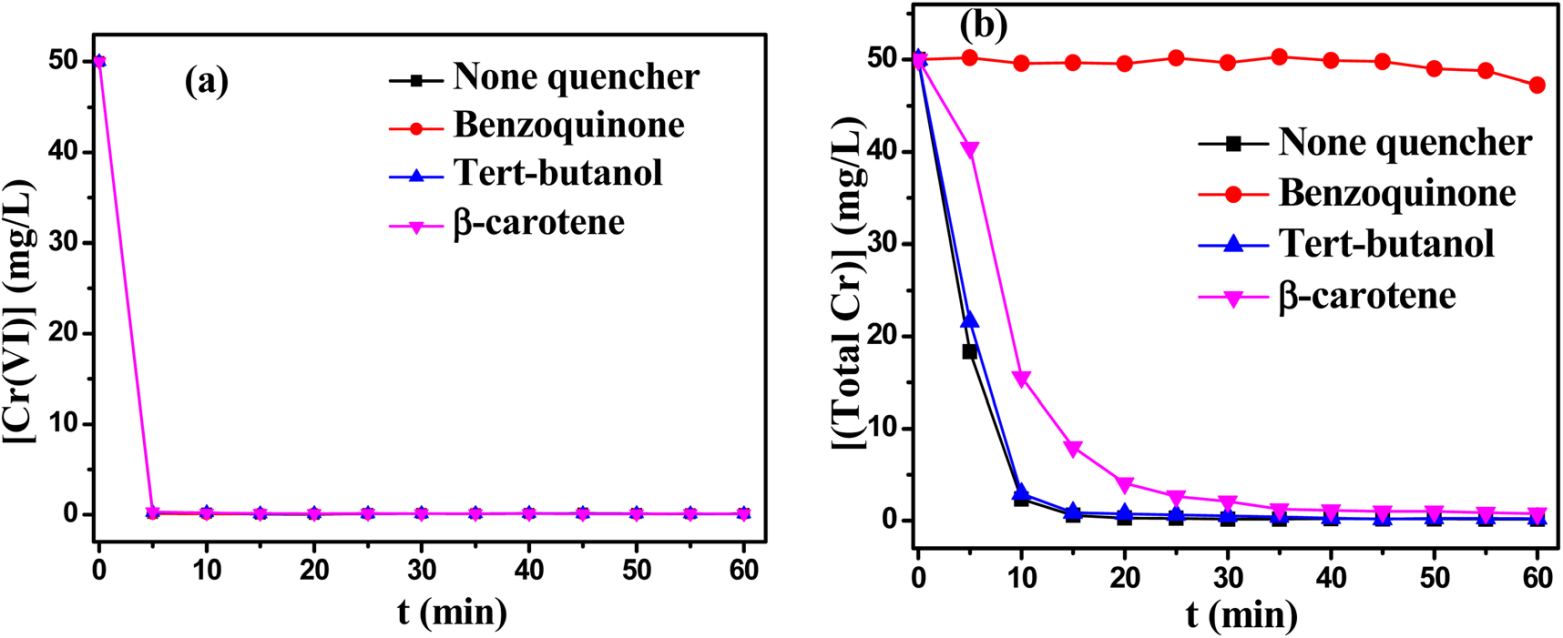
Effect of quenching agents on (a) the reduction of Cr(vi) and (b) the removal of total Cr. Reaction condition: [Cr(vi)] = 50 mg L^−1^, Cr(vi) : TD : MEA = 1 : 3 : 1, the initial pH value of 12, temperature 60 °C.

Further, quenching experiments were conducted to elucidate the contribution of reactive oxidants to the reduction/precipitation process. The effects of various quenching agents on the removal of Cr(vi) and total Cr from solution were investigated. As shown in Fig. 9(a), the experimental results revealed that reactive oxidants had no influence on the reduction process due to the unchanged removal rate of Cr(vi). However, Fig. 9(b) displayed that the removal of total Cr might change obviously in the presence of different quenching agents. When *p*-benzoquinone (BQ, 5 mM) was added as quenching agent for O_2_˙^−^ radical, the removal of total Cr became very low. It might be explained by the fact that the Cr–TD complex was efficiently decomposed by O_2_˙^−^ radical.^38–40^ In other words, the generation of Cr(OH)_3_ precipitates was inhibited in the absence of O_2_˙^−^ radical, resulting in the decrease of the removal of total Cr. Meanwhile, the precipitates of Cr(OH)_3_ was also obviously suppressed with adding β-carotene as a quench agent for ^1^O_2_,^41^ which proved that ^1^O_2_ also promoted the decomposition of Cr–TD complex to a great extent. *tert*-Butanol (TBA) was used as the quenching agent for HO˙. The removal of chromium ions almost unchanged in the presence of TBA, it also proved that there was no HO˙ radicals in this reaction system.

### 3.5 Practical wastewater application

**Fig. 10:**
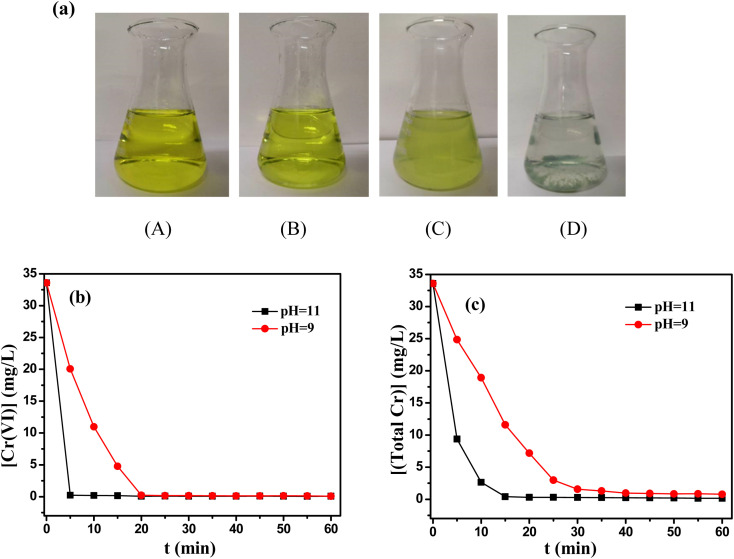
(a) Color variations of solution during reduction/precipitation with [Cr(vi)] : [TD] : [MEA] = 1 : 3 : 1. (A) Raw wastewater; (B) color of solution after adjusting pH to 9; (C) color of solution after 5 min; (D) precipitation phenomenon after 1 h. (b) The reduction of Cr(vi) in practical industrial wastewater at pH 9 and 11; (c) the removal of total Cr from in practical industrial wastewater at pH 9 and 11.

Chromium containing wastewater from Wuhan Iron and Steel (Group) Company was collected and used as practical industrial wastewater, which mainly came from chromate passivation process. The main parameters of chromium containing wastewater were the initial concentration of Cr(vi) 33.58 mg L^−1^ and initial pH 5.9. To reveal the change of chromium ions during reaction process, the color change of solution was presented in Fig. 10(a). It could be found that the yellow color of solution faded and the solution became cloudy after adding TD for 5 minutes. At last, the green precipitate of Cr(OH)_3_ settled on the bottom of the bottle. Correspondingly, the content of Cr(vi) decreased dramatically from 33.58 mg L^−1^ to 0.22, 0.079 mg L^−1^ after 20 minutes at pH 9 and 11, respectively (Fig. 10(b)), and then the total Cr decreased to 0.71, 0.16 mg L^−1^ after 1 h (Fig. 10(c)), which also met discharge standards for chromium-containing wastewater.

## 4. Conclusion

A green single TD and two-component reducing agents (TD/MEA) for the removal of Cr(vi) were first presented in this paper. The reduction/precipitation could occur simultaneously in the range of pH 8–12 in the presence of two-component reducing agents. Reductive and oxidative reactive species were produced simultaneously in this reaction system. The reactive oxidants (O_2_˙^−^ and ^1^O_2_) enhanced decomplexation of Cr(iii)–TD and promoted the formation of Cr(iii) precipitation. For two-component TD/MEA reaction system, amine exchange reaction between TD and MEA was confirmed, which enhanced reducibility of TD by facilitating the movement of the reaction equilibrium of tandem reaction. Especially, the reduction/precipitation occurred under alkaline conditions simultaneously, the final pH value of the solution returned to nearly neutral after the reaction, hence the amount of acid and base decreased. The main products of the reaction were sulfate and urea, which would not cause secondary pollution. Practical chromium containing wastewater was treated with TD/MEA as two-component reducing agents, the treated wastewater met the national discharge standard, which revealed that TD/MEA were highly efficient, eco-friendly two-component reducing agents for the removal of Cr(vi) from aqueous solutions.

## Conflicts of interest

The authors declare that they have no known competing financial interests or personal relationships that could have appeared to influence the work reported in this paper.

## Supplementary Material

RA-013-D3RA00520H-s001
