# Supplementary material for: One-step removal of hexavalent chromium in wide pH range using thiourea dioxide: the role of reactive species
Source: RSC Adv. 2023 Apr 4;13(16):10693–702. doi: 10.1039/d3ra00520h (PMC10072199; doi:10.1039/d3ra00520h)
Supplement: RA-013-D3RA00520H-s001 [file RA-013-D3RA00520H-s001.pdf]

Electronic Supplementary Material (ESI) for RSC Advances.
This journal is © The Royal Society of Chemistry 2023

Supplementary Information

One-step removal of hexavalent chromium in wide pH range using thiourea dioxide: the role of reactive species

Bin Lei[a, 1], Chaoyang Wang[a, 1], Ran Zhang[a,b], ZhiYong Xue[a,b], Feifei Chen[*a,b]

[a] Hubei Key laboratory of Biomass Fibers and Eco-dyeing and Finishing, Wuhan Textile University, Wuhan, 430073, Hubei, china

[b] College of Chemistry and Chemical Engineering, Wuhan Textile University, Wuhan, 430073, Hubei, China

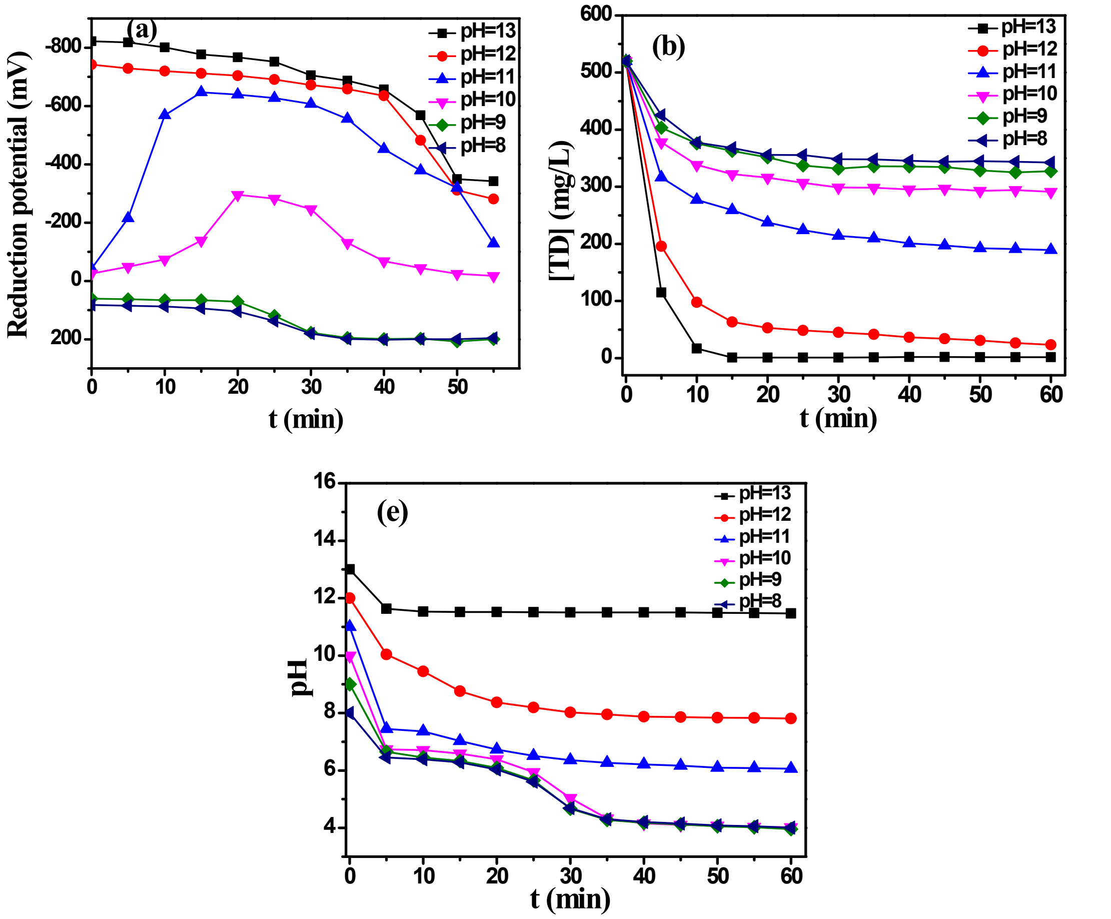

Fig.S1 Effects of the initial pH on (a) the change of reduction potential; (b) the decomposition rate of TD; (c) the change of pH. Reaction condition: T = 60°C, [Cr(VI)]/[TD] = 1:5.

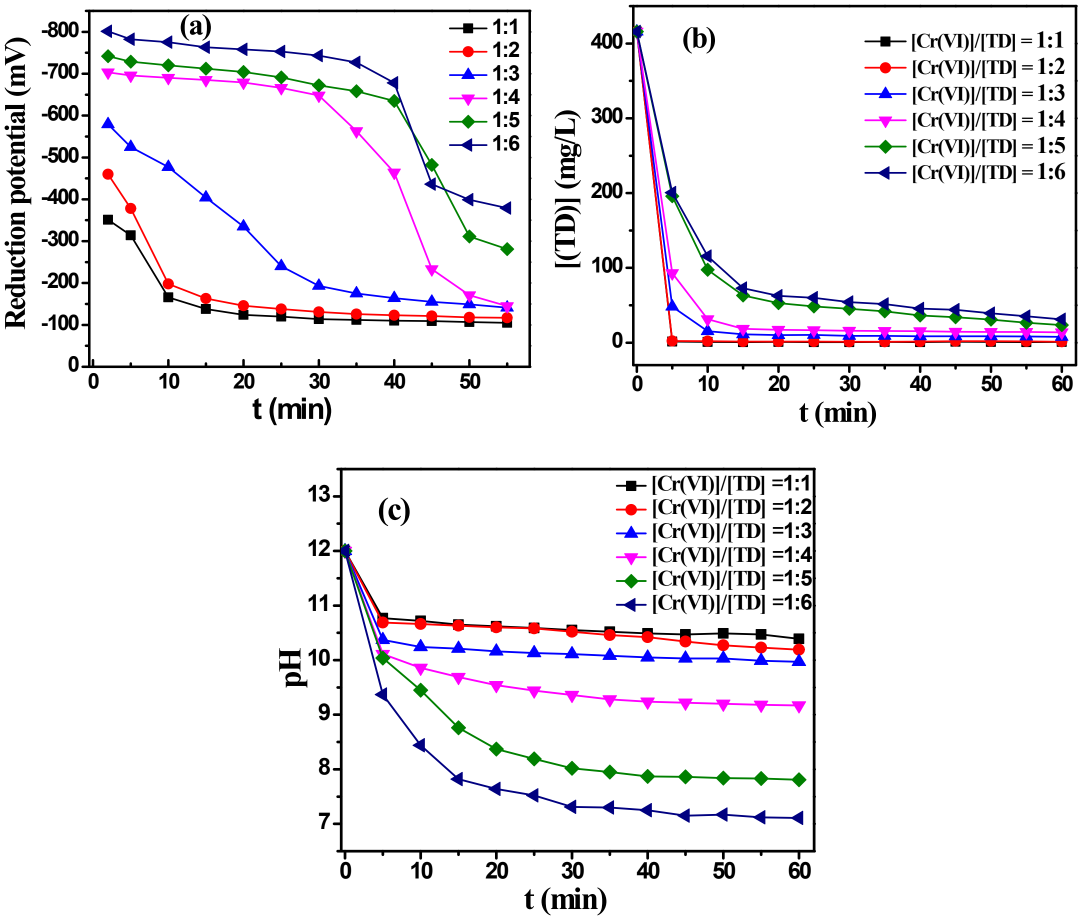

Fig.S2 Effects of TD dosage on (a) the change of reduction potential; (b) the decomposition rate of TD; (c) the change of pH. Reaction condition: T = 60°C, the initial pH value of 12, 50 mg/L Cr(VI) concentration .

Table.S1 Effect of nucleophilic reagent on the removal of Cr(VI) and total Cr

The second component	Residual Cr(VI) concentration (mg/L)	Residual total Cr concentration (mg/L)
Ethanolamine (ETA)	0.114	0.482
Diethanolamine (DEA)	0.098	0.519
Triethanolamine (TEOA)	0.121	49.73
Ethanol (EtOH)	49.24	49.85
Diethylenetriamine (DETA)	0.142	49.21
Trimethylamine (TEA)	0.163	0.432

Reaction conditions: the molar ratio of Cr(VI) : TD : X = 1: 4: 2, the initial pH value of 12, 50 mg/L

Cr(VI) concentration, temperature 60 °C, 1h.

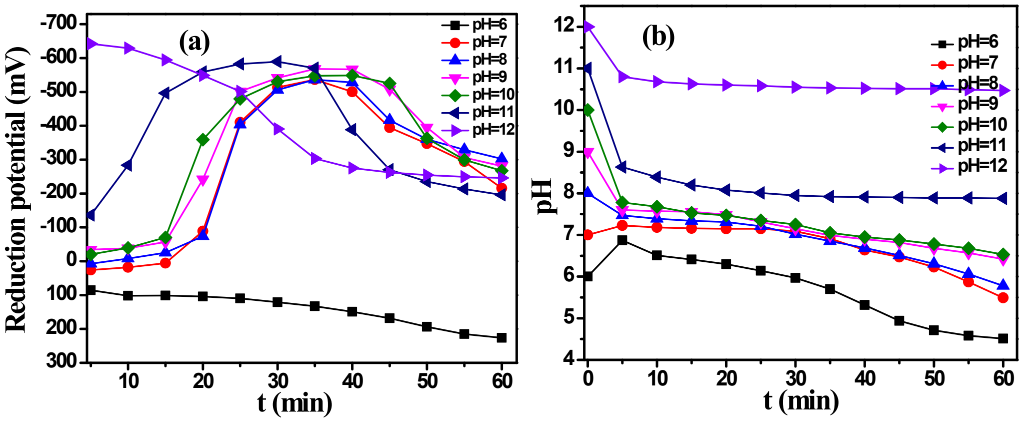

Fig.S3 Effect of the initial pH on (a) the change of reduction potential; (b) the change of pH. (Cr(VI): TD : ETA =1:3:1, [Cr(VI)] = 50 mg/L, temperature 60°C)

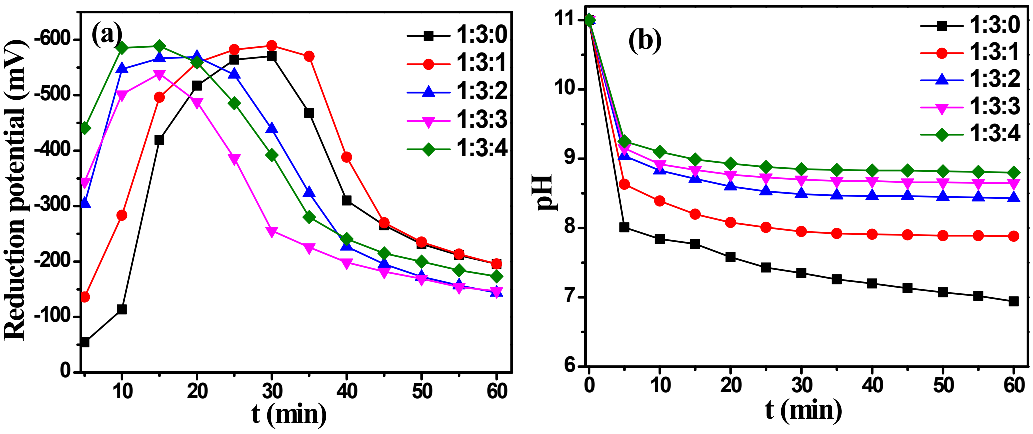

Fig.S4 Effect of the composing proportion of TD/ETA on (a) the change of reduction potential; (b) the change of pH. (T = 60°C, the initial pH value of 12, 50 mg/L Cr(VI) concentration).

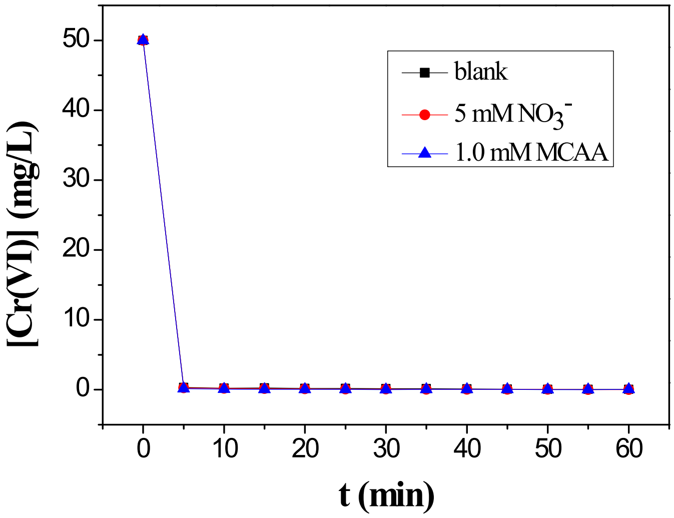

Fig.S5 Inhibitory effect of NO_3^- and MCAA on the removal of Cr(VI). (Cr(VI): TD : ETA =1:3:1, [Cr(VI)] = 50 mg/L, temperature 60°C)
